# Tetra­aqua­{1-[(1*H*-1,2,3-benzotriazol-1-yl)meth­yl]-1*H*-1,2,4-triazole}sulfato­cobalt(II) dihydrate

**DOI:** 10.1107/S1600536811027231

**Published:** 2011-07-13

**Authors:** Yu-xian Li, Da-wei Li, Dong Zhao

**Affiliations:** a Pharmacy College, Henan University of Traditional Chinese Medicine, Zhengzhou 450008, People’s Republic of China; b ZhengZhou Trade and Industry Schools, Zhengzhou 450000, People’s Republic of China; c Department of Chemistry, Zhengzhou University, Zhengzhou 450052, People’s Republic of China

## Abstract

In the title complex, [Co(SO_4_)(C_9_H_8_N_6_)(H_2_O)_4_]·2H_2_O, the Co^II^ ion is six-coordinated by one N atom from a 1*H*-1,2,3-benzotriazol-1-yl)meth­yl]-1*H*-1,2,4-triazole ligand, one O atom from a monodentate sulfate ligand and four water mol­ecules in a slightly distorted octa­hedral geometry. The sulfate ligand is rotationally disordered over two sets of sites with refined occupancies of 0.662 (15) and 0.338 (15). In the crystal, complex mol­ecules and solvent water mol­ecules are linked through inter­molecular O—H⋯O and O—H⋯N hydrogen bonds into a three-dimensional network.

## Related literature

For background to complexes constructed from *N*-heterocyclic ligands, see: Tian *et al.* (2010 ▶); Shi *et al.* (2010 ▶).
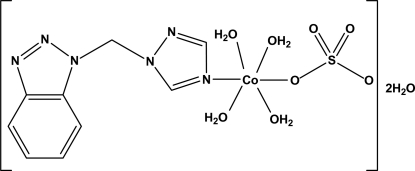


## Experimental

### 

#### Crystal data

[Co(SO_4_)(C_9_H_8_N_6_)(H_2_O)_4_]·2H_2_O
*M* *_r_* = 463.30
Triclinic, 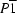

*a* = 7.5471 (15) Å
*b* = 7.9415 (16) Å
*c* = 16.198 (3) Å
α = 99.79 (3)°
β = 92.32 (3)°
γ = 112.22 (3)°
*V* = 879.8 (3) Å^3^
*Z* = 2
Mo *K*α radiation
μ = 1.16 mm^−1^
*T* = 293 K
0.21 × 0.19 × 0.16 mm

#### Data collection

Rigaku Saturn diffractometer
Absorption correction: multi-scan (*CrystalClear*; Rigaku/MSC, 2006 ▶) *T*_min_ = 0.793, *T*_max_ = 0.836
10814 measured reflections
4158 independent reflections
3868 reflections with *I* > 2σ(*I*)
*R*_int_ = 0.019

#### Refinement

*R*[*F* ^2^ > 2σ(*F* ^2^)] = 0.030
*wR*(*F* ^2^) = 0.069
*S* = 1.04
4158 reflections
272 parameters
H-atom parameters constrained
Δρ_max_ = 0.37 e Å^−3^
Δρ_min_ = −0.36 e Å^−3^

### 

Data collection: *CrystalClear* (Rigaku/MSC, 2006 ▶); cell refinement: *CrystalClear*; data reduction: *CrystalClear*; program(s) used to solve structure: *SHELXS97* (Sheldrick, 2008 ▶); program(s) used to refine structure: *SHELXL97* (Sheldrick, 2008 ▶); molecular graphics: *SHELXTL* (Sheldrick, 2008 ▶); software used to prepare material for publication: *SHELXTL*.

## Supplementary Material

Crystal structure: contains datablock(s) global, I. DOI: 10.1107/S1600536811027231/lh5272sup1.cif

Structure factors: contains datablock(s) I. DOI: 10.1107/S1600536811027231/lh5272Isup2.hkl

Additional supplementary materials:  crystallographic information; 3D view; checkCIF report

## Figures and Tables

**Table 1:** Hydrogen-bond geometry (Å, °)

*D*—H⋯*A*	*D*—H	H⋯*A*	*D*⋯*A*	*D*—H⋯*A*
O5—H1*W*⋯O4′	0.85	2.38	2.823 (13)	113
O5—H1*W*⋯N2^i^	0.85	2.31	3.081 (2)	150
O5—H2*W*⋯O10^ii^	0.85	1.83	2.672 (2)	173
O6—H3*W*⋯O9^iii^	0.85	1.95	2.798 (2)	171
O6—H4*W*⋯O2′^iv^	0.85	1.95	2.784 (7)	167
O6—H4*W*⋯O2^iv^	0.85	1.96	2.777 (4)	160
O7—H5*W*⋯O2^v^	0.85	1.94	2.771 (4)	167
O7—H5*W*⋯O2′^v^	0.85	2.15	2.961 (11)	159
O7—H6*W*⋯O9^vi^	0.85	1.89	2.728 (2)	168
O8—H8*W*⋯O3′^vi^	0.85	1.86	2.681 (7)	162
O8—H8*W*⋯O3^vi^	0.85	1.87	2.715 (3)	170
O8—H7*W*⋯O1^v^	0.85	1.99	2.8246 (18)	167
O9—H9*W*⋯O3^vii^	0.85	1.95	2.768 (6)	162
O9—H9*W*⋯O2′^vii^	0.85	2.19	2.911 (15)	142
O10—H11*W*⋯O4^vii^	0.85	1.97	2.809 (7)	168
O10—H11*W*⋯O4′^vii^	0.85	2.39	3.209 (16)	163
O10—H11*W*⋯O3′^vii^	0.85	2.39	2.996 (17)	129
O9—H10*W*⋯O1^viii^	0.85	2.11	2.945 (2)	166
O10—H12*W*⋯N6^ix^	0.85	2.00	2.853 (2)	177

Symmetry codes: (i) 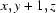; (ii) 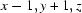; (iii) 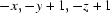; (iv) 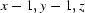; (v) 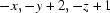; (vi) 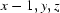; (vii) 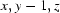; (viii) 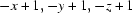; (ix) 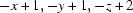.

**Fig. 1.**
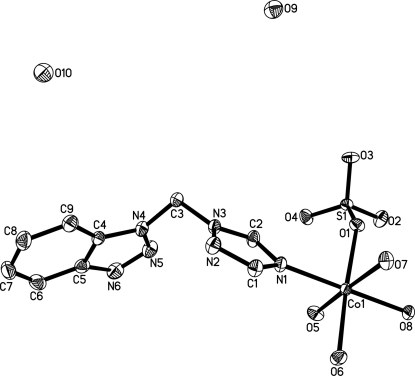
View of the asymmetric unit of the title complex with displacement ellipsoids displayed at the 30% probability level. H atoms are omitted for clarity. Only one component of the disordered SO4 ligand is shown.

**Fig. 2.**
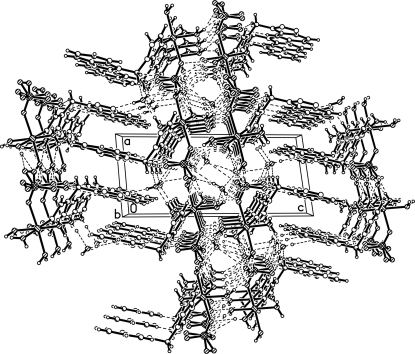
Part of the crystal structure with hydrogen bonds indicated by dashed lines.

## supplementary crystallographic information

### Comment

Numerous one-, two- and three dimensional complexes constructed from N-heterocyclic ligands have been synthesized (Tian *et al.*, 2010; Shi *et al.*, 2010). To further explore frameworks with new structures, we used 1*H*-1,2,3-benzotriazol-1-yl)methyl]-1*H*-1,2,4-triazole which has abundant N-donor sites to self-assembly with CoSO_4_ and obtained the title complex of which the crystal structure is reported herein. As shown in Figure 1, the Co^II^ ion is in a slightly distorted octahedral coordination environment defined by five oxygen atoms, four from water molecules and one from monodentate sulfate ligand and one nitrogen atom from a 1*H*-1,2,3-benzotriazol-1-yl)methyl]-1*H*-1,2,4-triazole ligand. Atoms O1, O5, O6, O7 and Co1 are essentially co-planar (the mean deviation from the plane is 0.0238 Å). Atom O8 and N1 atoms are located in the apical sites. The SO_4_ ligand is rotationally disordered about an S—O bond passing though atoms O1 and S1. In the crystal, complex molecules and solvent water molecules linked through intermolecular O—H···O and O—H···N hydrogen bonds into a three-dimensional network (Figure 2).

### Experimental

The ligand 1*H*-1,2,3-benzotriazol-1-yl)methyl]-1*H*-1,2,4-triazole (0.1 mmol) in methanol (4 ml) was added dropwise to an aqueous solution (2 ml) of cobalt sulfate (0.1 mmol). The resulting solution was allowed to stand at room temperature. After three weeks good quality red crystals were obtained from the filtrate and dried in air.

### Refinement

The disordered sulfate ligand was modeled by splitting the atoms into two components (O2, O3, O4 and O2', O3', O4'), the site occupation factors of which refined in a ratio of 0.662 (15):0.338 (15). H atoms are positioned geometrically and refined as riding atoms, with C-H = 0.93 (aromatic) and 0.97 (CH_2_) Å and O-H = 0.85 Å, and with U_iso_(H) = 1.2 U_eq_(C,O).

### Crystal data

**Table:**

[Co(SO_4_)(C_9_H_8_N_6_)(H_2_O)_4_]·2H_2_O	*Z* = 2
*M**_r_* = 463.30	*F*(000) = 478
Triclinic, *P*1	*D*_x_ = 1.749 Mg m^−^^3^
Hall symbol: -P 1	Mo *K*α radiation, λ = 0.71073 Å
*a* = 7.5471 (15) Å	Cell parameters from 3052 reflections
*b* = 7.9415 (16) Å	θ = 2.6–27.9°
*c* = 16.198 (3) Å	µ = 1.16 mm^−^^1^
α = 99.79 (3)°	*T* = 293 K
β = 92.32 (3)°	Prism, red
γ = 112.22 (3)°	0.21 × 0.19 × 0.16 mm
*V* = 879.8 (3) Å^3^	

### Data collection

**Table:**

Rigaku Saturn diffractometer	4158 independent reflections
Radiation source: fine-focus sealed tube	3868 reflections with *I* > 2σ(*I*)
graphite	*R*_int_ = 0.019
Detector resolution: 28.5714 pixels mm^-1^	θ_max_ = 27.9°, θ_min_ = 2.6°
ω scans	*h* = −9→9
Absorption correction: multi-scan (*CrystalClear*; Rigaku/MSC, 2006)	*k* = −10→10
*T*_min_ = 0.793, *T*_max_ = 0.836	*l* = −21→19
10814 measured reflections	

### Refinement

**Table:**

Refinement on *F*^2^	Primary atom site location: structure-invariant direct methods
Least-squares matrix: full	Secondary atom site location: difference Fourier map
*R*[*F*^2^ > 2σ(*F*^2^)] = 0.030	Hydrogen site location: inferred from neighbouring sites
*wR*(*F*^2^) = 0.069	H-atom parameters constrained
*S* = 1.04	*w* = 1/[σ^2^(*F*_o_^2^) + (0.0299*P*)^2^ + 0.4582*P*] where *P* = (*F*_o_^2^ + 2*F*_c_^2^)/3
4158 reflections	(Δ/σ)_max_ = 0.001
272 parameters	Δρ_max_ = 0.37 e Å^−^^3^
0 restraints	Δρ_min_ = −0.36 e Å^−^^3^

### Special details

**Table:**

Geometry. All e.s.d.'s (except the e.s.d. in the dihedral angle between two l.s. planes) are estimated using the full covariance matrix. The cell e.s.d.'s are taken into account individually in the estimation of e.s.d.'s in distances, angles and torsion angles; correlations between e.s.d.'s in cell parameters are only used when they are defined by crystal symmetry. An approximate (isotropic) treatment of cell e.s.d.'s is used for estimating e.s.d.'s involving l.s. planes.
Refinement. Refinement of *F*^2^ against ALL reflections. The weighted *R*-factor *wR* and goodness of fit *S* are based on *F*^2^, conventional *R*-factors *R* are based on *F*, with *F* set to zero for negative *F*^2^. The threshold expression of *F*^2^ > σ(*F*^2^) is used only for calculating *R*-factors(gt) *etc*. and is not relevant to the choice of reflections for refinement. *R*-factors based on *F*^2^ are statistically about twice as large as those based on *F*, and *R*- factors based on ALL data will be even larger.

### Fractional atomic coordinates and isotropic or equivalent isotropic displacement parameters (Å^2^)

**Table:**

	*x*	*y*	*z*	*U*_iso_*/*U*_eq_	Occ. (<1)
Co1	−0.08883 (3)	0.83076 (3)	0.627602 (13)	0.02224 (7)	
N1	0.0089 (2)	0.65836 (19)	0.68655 (9)	0.0277 (3)	
N2	0.0484 (2)	0.4200 (2)	0.73017 (10)	0.0328 (3)	
N3	0.1979 (2)	0.58276 (19)	0.76299 (9)	0.0267 (3)	
N4	0.3108 (2)	0.5942 (2)	0.90460 (9)	0.0305 (3)	
N5	0.3118 (3)	0.7536 (2)	0.95135 (11)	0.0401 (4)	
N6	0.2705 (3)	0.7243 (3)	1.02609 (11)	0.0426 (4)	
O1	0.19941 (16)	0.98544 (16)	0.60342 (7)	0.0298 (3)	
O2	0.3506 (7)	1.3182 (4)	0.6265 (3)	0.0383 (9)	0.662 (15)
O3	0.5396 (3)	1.1350 (6)	0.6050 (3)	0.0360 (11)	0.662 (15)
O4	0.4014 (9)	1.1668 (11)	0.7346 (4)	0.0379 (10)	0.662 (15)
O2'	0.4176 (19)	1.2967 (10)	0.6045 (5)	0.047 (2)	0.338 (15)
O3'	0.5343 (8)	1.0771 (14)	0.6418 (9)	0.060 (3)	0.338 (15)
O4'	0.3471 (18)	1.181 (2)	0.7357 (9)	0.045 (3)	0.338 (15)
O5	−0.05411 (19)	1.01086 (18)	0.74101 (8)	0.0364 (3)	
H1W	0.0162	1.1247	0.7437	0.044*	
H2W	−0.1552	1.0065	0.7635	0.044*	
O6	−0.38083 (17)	0.68003 (17)	0.64472 (9)	0.0357 (3)	
H3W	−0.4664	0.6946	0.6144	0.043*	
H4W	−0.4390	0.5635	0.6404	0.043*	
O7	−0.11655 (19)	0.67039 (17)	0.50698 (8)	0.0334 (3)	
H5W	−0.1783	0.6924	0.4680	0.040*	
H6W	−0.1752	0.5533	0.4998	0.040*	
O8	−0.18197 (18)	1.00403 (18)	0.57005 (8)	0.0324 (3)	
H7W	−0.1737	1.0251	0.5203	0.039*	
H8W	−0.2657	1.0454	0.5869	0.039*	
O9	0.65865 (19)	0.29975 (18)	0.46850 (9)	0.0384 (3)	
H9W	0.6003	0.2531	0.5081	0.046*	
H10W	0.7165	0.2280	0.4539	0.046*	
O10	0.6405 (2)	0.0290 (2)	0.81395 (9)	0.0430 (3)	
H11W	0.5841	0.0841	0.7891	0.052*	
H12W	0.6696	0.1011	0.8620	0.052*	
C1	−0.0606 (3)	0.4727 (2)	0.68424 (11)	0.0320 (4)	
H1A	−0.1758	0.3899	0.6528	0.038*	
C2	0.1713 (3)	0.7220 (2)	0.73708 (11)	0.0321 (4)	
H2A	0.2548	0.8462	0.7523	0.039*	
C3	0.3579 (3)	0.5914 (3)	0.81927 (11)	0.0311 (4)	
H3A	0.3893	0.4845	0.8002	0.037*	
H3B	0.4703	0.7022	0.8176	0.037*	
C4	0.2692 (2)	0.4584 (2)	0.95100 (10)	0.0279 (3)	
C5	0.2429 (3)	0.5440 (3)	1.02937 (11)	0.0345 (4)	
C6	0.2012 (3)	0.4478 (4)	1.09590 (13)	0.0476 (5)	
H6A	0.1838	0.5035	1.1486	0.057*	
C7	0.1874 (3)	0.2690 (4)	1.07968 (15)	0.0510 (6)	
H7A	0.1579	0.2006	1.1221	0.061*	
C8	0.2164 (3)	0.1848 (3)	1.00044 (15)	0.0458 (5)	
H8A	0.2077	0.0630	0.9926	0.055*	
C9	0.2573 (3)	0.2761 (3)	0.93436 (12)	0.0367 (4)	
H9A	0.2757	0.2201	0.8820	0.044*	
S1	0.37497 (5)	1.14888 (5)	0.64536 (2)	0.02329 (9)	

### Atomic displacement parameters (Å^2^)

**Table:**

	*U*^11^	*U*^22^	*U*^33^	*U*^12^	*U*^13^	*U*^23^
Co1	0.02054 (11)	0.02314 (12)	0.02269 (11)	0.00744 (8)	0.00097 (8)	0.00663 (8)
N1	0.0270 (7)	0.0262 (7)	0.0292 (7)	0.0083 (6)	−0.0018 (5)	0.0096 (6)
N2	0.0346 (8)	0.0230 (7)	0.0377 (8)	0.0087 (6)	−0.0027 (6)	0.0059 (6)
N3	0.0260 (7)	0.0268 (7)	0.0258 (7)	0.0081 (6)	−0.0012 (5)	0.0077 (6)
N4	0.0361 (8)	0.0314 (8)	0.0263 (7)	0.0165 (6)	−0.0014 (6)	0.0048 (6)
N5	0.0495 (10)	0.0360 (9)	0.0374 (9)	0.0231 (8)	−0.0025 (7)	0.0007 (7)
N6	0.0487 (10)	0.0470 (10)	0.0352 (9)	0.0268 (8)	0.0002 (7)	−0.0022 (7)
O1	0.0213 (6)	0.0294 (6)	0.0287 (6)	0.0002 (5)	0.0023 (5)	0.0026 (5)
O2	0.0438 (18)	0.0245 (11)	0.0470 (19)	0.0144 (11)	−0.0031 (13)	0.0072 (11)
O3	0.0199 (10)	0.0397 (17)	0.0452 (19)	0.0098 (9)	0.0083 (9)	0.0035 (12)
O4	0.035 (3)	0.0462 (16)	0.0235 (14)	0.007 (2)	−0.0014 (18)	0.0060 (11)
O2'	0.065 (5)	0.024 (3)	0.042 (3)	0.004 (3)	−0.002 (3)	0.014 (2)
O3'	0.033 (3)	0.069 (4)	0.092 (7)	0.032 (3)	0.017 (3)	0.020 (5)
O4'	0.032 (5)	0.054 (5)	0.027 (3)	−0.001 (4)	0.001 (4)	−0.007 (3)
O5	0.0345 (7)	0.0347 (7)	0.0300 (6)	0.0054 (5)	0.0062 (5)	−0.0008 (5)
O6	0.0229 (6)	0.0309 (6)	0.0502 (8)	0.0048 (5)	0.0015 (5)	0.0144 (6)
O7	0.0392 (7)	0.0267 (6)	0.0277 (6)	0.0079 (5)	−0.0035 (5)	0.0016 (5)
O8	0.0371 (7)	0.0403 (7)	0.0308 (6)	0.0240 (6)	0.0061 (5)	0.0145 (5)
O9	0.0391 (7)	0.0298 (7)	0.0475 (8)	0.0140 (6)	0.0062 (6)	0.0093 (6)
O10	0.0486 (8)	0.0457 (8)	0.0340 (7)	0.0208 (7)	0.0026 (6)	0.0012 (6)
C1	0.0301 (8)	0.0264 (8)	0.0340 (9)	0.0061 (7)	−0.0040 (7)	0.0053 (7)
C2	0.0313 (9)	0.0258 (8)	0.0349 (9)	0.0053 (7)	−0.0044 (7)	0.0109 (7)
C3	0.0282 (8)	0.0399 (10)	0.0276 (8)	0.0149 (7)	0.0000 (7)	0.0103 (7)
C4	0.0252 (8)	0.0340 (9)	0.0251 (8)	0.0123 (7)	−0.0012 (6)	0.0068 (7)
C5	0.0313 (9)	0.0442 (10)	0.0290 (9)	0.0177 (8)	0.0001 (7)	0.0041 (8)
C6	0.0420 (11)	0.0752 (16)	0.0294 (9)	0.0247 (11)	0.0090 (8)	0.0155 (10)
C7	0.0398 (11)	0.0680 (15)	0.0478 (12)	0.0146 (10)	0.0056 (9)	0.0334 (12)
C8	0.0406 (11)	0.0392 (11)	0.0588 (13)	0.0121 (9)	0.0013 (10)	0.0221 (10)
C9	0.0361 (10)	0.0348 (10)	0.0382 (10)	0.0138 (8)	−0.0006 (8)	0.0061 (8)
S1	0.01968 (18)	0.02186 (19)	0.02462 (19)	0.00521 (14)	0.00123 (14)	0.00217 (15)

### Geometric parameters (Å, °)

**Table:**

Co1—O5	2.0672 (15)	O5—H2W	0.8500
Co1—O8	2.0907 (13)	O6—H3W	0.8500
Co1—O7	2.0982 (14)	O6—H4W	0.8500
Co1—N1	2.1169 (15)	O7—H5W	0.8501
Co1—O6	2.1292 (14)	O7—H6W	0.8500
Co1—O1	2.1462 (14)	O8—H7W	0.8498
N1—C2	1.318 (2)	O8—H8W	0.8500
N1—C1	1.358 (2)	O9—H9W	0.8500
N2—C1	1.312 (2)	O9—H10W	0.8500
N2—N3	1.357 (2)	O10—H11W	0.8500
N3—C2	1.326 (2)	O10—H12W	0.8500
N3—C3	1.456 (2)	C1—H1A	0.9300
N4—N5	1.357 (2)	C2—H2A	0.9300
N4—C4	1.365 (2)	C3—H3A	0.9700
N4—C3	1.440 (2)	C3—H3B	0.9700
N5—N6	1.300 (2)	C4—C5	1.393 (2)
N6—C5	1.378 (3)	C4—C9	1.394 (3)
O1—S1	1.4927 (14)	C5—C6	1.403 (3)
O2—S1	1.503 (3)	C6—C7	1.362 (4)
O3—S1	1.459 (2)	C6—H6A	0.9300
O4—S1	1.426 (7)	C7—C8	1.409 (3)
O2'—S1	1.384 (6)	C7—H7A	0.9300
O3'—S1	1.512 (6)	C8—C9	1.374 (3)
O4'—S1	1.480 (14)	C8—H8A	0.9300
O5—H1W	0.8500	C9—H9A	0.9300
			
O5—Co1—O8	87.68 (6)	N3—C2—H2A	125.0
O5—Co1—O7	174.46 (5)	N4—C3—N3	111.19 (14)
O8—Co1—O7	87.68 (5)	N4—C3—H3A	109.4
O5—Co1—N1	91.89 (6)	N3—C3—H3A	109.4
O8—Co1—N1	179.24 (5)	N4—C3—H3B	109.4
O7—Co1—N1	92.72 (6)	N3—C3—H3B	109.4
O5—Co1—O6	90.03 (6)	H3A—C3—H3B	108.0
O8—Co1—O6	88.17 (6)	N4—C4—C5	103.75 (16)
O7—Co1—O6	92.85 (6)	N4—C4—C9	133.59 (17)
N1—Co1—O6	92.46 (6)	C5—C4—C9	122.63 (17)
O5—Co1—O1	91.77 (6)	N6—C5—C4	108.34 (16)
O8—Co1—O1	89.00 (6)	N6—C5—C6	130.85 (19)
O7—Co1—O1	85.13 (6)	C4—C5—C6	120.79 (19)
N1—Co1—O1	90.39 (6)	C7—C6—C5	116.8 (2)
O6—Co1—O1	176.59 (5)	C7—C6—H6A	121.6
C2—N1—C1	103.18 (14)	C5—C6—H6A	121.6
C2—N1—Co1	123.09 (12)	C6—C7—C8	121.9 (2)
C1—N1—Co1	133.72 (12)	C6—C7—H7A	119.1
C1—N2—N3	102.41 (14)	C8—C7—H7A	119.1
C2—N3—N2	110.20 (14)	C9—C8—C7	122.3 (2)
C2—N3—C3	127.96 (15)	C9—C8—H8A	118.8
N2—N3—C3	121.83 (14)	C7—C8—H8A	118.8
N5—N4—C4	110.80 (15)	C8—C9—C4	115.56 (19)
N5—N4—C3	119.16 (15)	C8—C9—H9A	122.2
C4—N4—C3	129.99 (16)	C4—C9—H9A	122.2
N6—N5—N4	108.13 (16)	O2'—S1—O4	124.5 (5)
N5—N6—C5	108.98 (16)	O2'—S1—O3	80.7 (5)
S1—O1—Co1	139.41 (8)	O4—S1—O3	112.5 (2)
Co1—O5—H1W	117.2	O2'—S1—O4'	117.9 (7)
Co1—O5—H2W	117.5	O4—S1—O4'	18.4 (5)
H1W—O5—H2W	107.3	O3—S1—O4'	130.4 (4)
Co1—O6—H3W	116.6	O2'—S1—O1	112.5 (3)
Co1—O6—H4W	128.0	O4—S1—O1	113.3 (3)
H3W—O6—H4W	96.6	O3—S1—O1	107.70 (11)
Co1—O7—H5W	115.8	O4'—S1—O1	105.7 (6)
Co1—O7—H6W	118.4	O2'—S1—O2	27.1 (5)
H5W—O7—H6W	100.2	O4—S1—O2	108.7 (3)
Co1—O8—H7W	130.1	O3—S1—O2	107.59 (16)
Co1—O8—H8W	125.4	O4'—S1—O2	96.4 (6)
H7W—O8—H8W	102.5	O1—S1—O2	106.69 (14)
H9W—O9—H10W	100.8	O2'—S1—O3'	110.4 (4)
H11W—O10—H12W	98.1	O4—S1—O3'	86.7 (5)
N2—C1—N1	114.30 (15)	O3—S1—O3'	31.4 (4)
N2—C1—H1A	122.9	O4'—S1—O3'	104.9 (5)
N1—C1—H1A	122.9	O1—S1—O3'	104.3 (3)
N1—C2—N3	109.90 (15)	O2—S1—O3'	135.6 (4)
N1—C2—H2A	125.0		
			
O5—Co1—N1—C2	−51.28 (15)	N5—N4—C3—N3	−76.3 (2)
O8—Co1—N1—C2	4(4)	C4—N4—C3—N3	106.5 (2)
O7—Co1—N1—C2	125.64 (15)	C2—N3—C3—N4	96.6 (2)
O6—Co1—N1—C2	−141.39 (15)	N2—N3—C3—N4	−82.3 (2)
O1—Co1—N1—C2	40.50 (15)	N5—N4—C4—C5	0.45 (19)
O5—Co1—N1—C1	130.32 (17)	C3—N4—C4—C5	177.87 (17)
O8—Co1—N1—C1	−174 (100)	N5—N4—C4—C9	−177.36 (19)
O7—Co1—N1—C1	−52.76 (17)	C3—N4—C4—C9	0.1 (3)
O6—Co1—N1—C1	40.21 (17)	N5—N6—C5—C4	−0.1 (2)
O1—Co1—N1—C1	−137.90 (17)	N5—N6—C5—C6	178.1 (2)
C1—N2—N3—C2	0.9 (2)	N4—C4—C5—N6	−0.20 (19)
C1—N2—N3—C3	−179.95 (16)	C9—C4—C5—N6	177.92 (17)
C4—N4—N5—N6	−0.6 (2)	N4—C4—C5—C6	−178.62 (17)
C3—N4—N5—N6	−178.29 (15)	C9—C4—C5—C6	−0.5 (3)
N4—N5—N6—C5	0.4 (2)	N6—C5—C6—C7	−178.2 (2)
O5—Co1—O1—S1	3.70 (13)	C4—C5—C6—C7	−0.2 (3)
O8—Co1—O1—S1	91.36 (12)	C5—C6—C7—C8	1.0 (3)
O7—Co1—O1—S1	179.11 (13)	C6—C7—C8—C9	−1.1 (3)
N1—Co1—O1—S1	−88.19 (13)	C7—C8—C9—C4	0.3 (3)
O6—Co1—O1—S1	125.4 (8)	N4—C4—C9—C8	177.90 (19)
N3—N2—C1—N1	−0.7 (2)	C5—C4—C9—C8	0.4 (3)
C2—N1—C1—N2	0.2 (2)	Co1—O1—S1—O2'	−113.2 (8)
Co1—N1—C1—N2	178.85 (13)	Co1—O1—S1—O4	34.7 (3)
C1—N1—C2—N3	0.4 (2)	Co1—O1—S1—O3	159.7 (3)
Co1—N1—C2—N3	−178.43 (11)	Co1—O1—S1—O4'	16.9 (7)
N2—N3—C2—N1	−0.9 (2)	Co1—O1—S1—O2	−85.0 (3)
C3—N3—C2—N1	−179.91 (16)	Co1—O1—S1—O3'	127.2 (7)

### Hydrogen-bond geometry (Å, °)

**Table:**

*D*—H···*A*	*D*—H	H···*A*	*D*···*A*	*D*—H···*A*
O5—H1W···O4'	0.85	2.38	2.823 (13)	113.
O5—H1W···N2^i^	0.85	2.31	3.081 (2)	150.
O5—H2W···O10^ii^	0.85	1.83	2.672 (2)	173.
O6—H3W···O9^iii^	0.85	1.95	2.798 (2)	171.
O6—H4W···O2'^iv^	0.85	1.95	2.784 (7)	167.
O6—H4W···O2^iv^	0.85	1.96	2.777 (4)	160.
O7—H5W···O2^v^	0.85	1.94	2.771 (4)	167.
O7—H5W···O2'^v^	0.85	2.15	2.961 (11)	159.
O7—H6W···O9^vi^	0.85	1.89	2.728 (2)	168.
O8—H8W···O3'^vi^	0.85	1.86	2.681 (7)	162.
O8—H8W···O3^vi^	0.85	1.87	2.715 (3)	170.
O8—H7W···O1^v^	0.85	1.99	2.8246 (18)	167.
O9—H9W···O3^vii^	0.85	1.95	2.768 (6)	162.
O9—H9W···O2'^vii^	0.85	2.19	2.911 (15)	142.
O10—H11W···O4^vii^	0.85	1.97	2.809 (7)	168.
O10—H11W···O4'^vii^	0.85	2.39	3.209 (16)	163.
O10—H11W···O3'^vii^	0.85	2.39	2.996 (17)	129.
O9—H10W···O1^viii^	0.85	2.11	2.945 (2)	166.
O10—H12W···N6^ix^	0.85	2.00	2.853 (2)	177.

Symmetry codes: (i) *x*, *y*+1, *z*; (ii) *x*−1, *y*+1, *z*; (iii) −*x*, −*y*+1, −*z*+1; (iv) *x*−1, *y*−1, *z*; (v) −*x*, −*y*+2, −*z*+1; (vi) *x*−1, *y*, *z*; (vii) *x*, *y*−1, *z*; (viii) −*x*+1, −*y*+1, −*z*+1; (ix) −*x*+1, −*y*+1, −*z*+2.
